# The Prevalence of Allergic Rhinitis in Patients with Chronic Rhinosinusitis 

**Published:** 2014-10

**Authors:** Mehdi Bakhshaee, Farahzad Jabari, Mohammad Mehdi Ghassemi, Shiva Hourzad, Russell Deutscher, Kianoosh Nahid

**Affiliations:** 1 *Sinus and Surgical Endoscopic Research Center, Ghaem Hospital, Faculty of Medicine, Mashhad University of Medical Sciences, Mashhad, Iran.*; 2 *Allergy Research Center, School of Medicine, Mashhad University of Medical Sciences, Mashhad, Iran.*; 3 *Medical Student, Faculty of Medicine, Mashhad University of Medical Sciences, Mashhad, Iran. *; 4 *Medical Student, Caribbean Medical University, Curacao. *; 5 **Department of Otorhinolaryngology, Mashhad University of Medical Sciences, Mashhad, Iran.**

**Keywords:** Allergy, Chronic rhinosinusitis, Polyposis, Skin prick test

## Abstract

**Introduction:**

Chronic rhinosinusitis (CRS) is a multifactorial disease. Allergies are considered a predisposing factor to CRS; however, this remains controversial. The objective of this research was to investigate the prevalence of co-morbidities and allergic reaction, and to specify the most common allergens in patients with confirmed CRS.

**Materials and Methods:**

One hundred patients with signs and symptoms of CRS who met the diagnostic endoscopic and radiologic criteria of chronic rhinosinusitis were selected. They filled out a questionnaire and underwent a skin prick test for the common inhalant allergens. Allergic rhinitis was diagnosed according to the history and positive skin prick tests.

**Results:**

The mean age of patients was 34. Males were slightly more involved (54%). The prevalence of polypoid and none-polypoid rhinosinusitis was 54% and 46% respectively. The patients’ most common symptoms were nasal discharge (95%), blockage (94%), smell disorders (63%), cough (45%), halitosis (41%), lethargy (37%), and aural fullness (36%). Allergy to at least one allergen was noted in 64% of the CRS patients which is higher than general population in Mashhad, Iran with allergic rhinitis (22.4%). Salsola was the most common allergen. There was no significant difference in allergic reactions between polypoid and non-polypoid CRS patients.

**Conclusion:**

Allergic reactions was found in Iranian CRS patients with or without polyposis to be much higher than general population in Mashhad with allergic rhinitis alone.

## Introduction

Chronic rhinosinusitis (CRS) is described as a multifactorial disease (took out comma) which causes chronic inflammation of the mucosa of the nasal cavity and paranasal sinuses, with a duration of more than 12 weeks. It involves at least 30% of the population and its pathophysiology is not completely understood. It is speculated that viral, bacterial, and fungal immunologic responses, along with the formation of biofilm are responsible for CRS. The following are believed to be predisposing factors for its development: allergies, anatomic variations in the nasal cavity and paranasal sinuses (concha bullosa, paradoxical turbinate), congenital disorders (immotile cilia syndrome, cystic fibrosis), some neoplasms and exposure to tobacco. The symptoms of CRS can be categorized into major and minor symptoms, as shown in (Table. 1).

**Table 1:** Major and minor symptoms/signs in CRS

**Major symptoms/signs**	**Minor symptoms/signs**
Facial pain/pressure	Tooth ache
Nasal blockage	Aural fullness/ pain/pressure
Nasal mucoid or purulent discharge	Lethargy
Post nasal discharge	Fever
Hyposmia	Halitosis
Anosmia	
Pus in nasal cavity in examination	

Allergies facilitate the progression of CRS, although, the actual mechanism is not fully understood. Allergic reactions among CRS patients vary between 25-75% geographically, which is higher than the non- CRS population (2-4). 

## Materials and Methods

This cross-sectional study was performed on 100 consecutive patients who referred to the ENT ambulatory unit of Mashhad University of Medical Sciences (MUMS, Mashhad, Iran) during the year 2012. The patients were diagnosed with CRS by the senior author according to the clinical, endoscopic, and radiologic diagnostic criteria of Rhinosinusitis Symptom Inventory (RSI). For ethical purposes, candidates were informed of the study and gave their consent before undergoing physical examination and skin prick tests. Their identities were kept confidential and the procedure was free of charge. The patient was excluded from the study if CRS could not be confirmed or the questionnaire was incomplete. Nasal polyps were detected by nasal endoscopy and classified into grades 1,2, and 3. Polyps restricted to the middle meatus were scored as grade 1 or mild, Polyps extending beyond the middle turbinate were scored as grade 2 or moderate, Polyps that filled the nasal cavity or extended to or beyond the inferior turbinate were scored as grade 3 or severe (5).The following were measures of outcome: The distribution of age and sex, frequency of signs, and major and minor symptoms, the prevalence of allergies and common allergens, the prevalence of asthma and smoking habit. 

After completion of the questionnaire and data-sheet for physical examination, endoscopic findings were obtained and the result of skin prick test for most usual allergens (Greer Co, USA) was obtained, the data was analyzed by SPSS version 13.0, and P value was set to 0.05(3). 

## Results

 One hundred patients, 54% males and 46% females, with chronic rhinosinusitis were included in this study. The subjects aged 14 to 65 years, with the mean age of 34.06±12.32 years. Nasal polyposis was seen in 54% of males and 35% of females diagnosed with CRS.

Non-polypoid and polypoid sinusitis were seen in 46% and 54% of cases respectively. Most cases of polyposis were severe (Table. 2). The most common presenting symptom was nasal blockage (62%). The patients reported the following symptoms: rhinorrhea (95%), nasal blockage (94%), smell disorders (63%), cough (45%), halitosis (41%), lethargy (37%) and aural fullness (36%).

**Table 2:** The prevalence polypoid vs none polypoid CRS and the severity

**CRS Type**	**Frequency**	**Percent**
None Polypoid	46	46%
Polypoid Total	54	54&
Severe	39	72.2%
Moderate	9	16.6%
Mild	6	11.1%

Prevalence of allergies among our subjects was 64% in total, 67% and 60% in patients with or without polyposis respectively. This difference was not statistically significant (P>0.5). There was also no significant difference in allergic response between males and females (P>0.5) and among different age groups (P>0.5). The most common allergens were found to be Salsola (Table. 3). 

**Table 3:** The most common allergens among patients with chronic rhinosinusitis

**Allergen**	**Frequency**
Tree.mixture	30%
Salsala	43%
Grasses	28%
Dermatophagoides.Ptronyssinus	14%
Dermatophagoides.Farinae	14%
Cockroach	10%
Aspergillus.mix	6%
Feather.mixture	3%
Altenaria	10%
Cat	4%

## Discussion

CRS is reported to be more common in the 6^th^ decade of life (6); however, the average age of our subjects was 34 years. 54% of our cases diagnosed with CRS had nasal polyposis while other similar studies reported it to be up to 70% (6,7). The prevalence of positive skin prick test result was 64%, which was similar to the results reported by Turkish and American studies and higher than the results reported by Polish and Italian studies (4,8-10). However, there was no statistically significant difference between polyposis and non-polyposis group; this is in accordance with research studies conducted in Italy and different from the results reported by Turkish and Lithuanian research studies (8,10,11). Although chronic rhinosinusitis with or without nasal polyps is often considered as one disease entity (12), CRS and nasal polyposis are distinct disease entities within the group of chronic sinus diseases due to differences in their inflammatory mediators (13).

Reaction to at least one allergen (64%) among patients with chronic rhinosinusitis was significantly higher than general population of our area (Mashhad, Iran) according to what Fereidouni et al was reported previously (22.4%) (14). Prevalence of allergies was not statistically different between males and females and also among different age groups. The studied carried out in Italy found the same results with regard to age.

The most common presenting complaint among our patients was nasal blockage (62%); we found no other similar study to compare.

Asthma was the most common cause of co-morbidity, which is in accordance with other studies. Asthma, on the other hand, was found to be more common in polyposis group, as discovered by the study performed in Italy. It is believed that asthma in severe CRS patients is related to eosinophilia of the nasal discharge. There is a strong relationship between severity of CRS and prevalence of asthma. Polyposis was also significantly more common among smokers in this study. The same result was reported by a study done in the US. 

Salasola, tree mixture, and grass were the most common allergens reported, while other research studies in the USA and Turkey have found mites to be the most common allergen. In Bangladesh, Perennial airborne allergens are reported to be the main pathogens for nasal polyposis (20). 

## Conclusion

Allergic response was common in the patients diagnosed with chronic rhinosinusitis in our study regardless of their age or sex. It seems allergic rhinitis is more common among patient with chronic rhinosinusitis compare to general population. The common allergens in our study (Salasola, tree mixture, and grass) differed from allergens reported in other countries. The high rate of positive allergic tests in the CRS patients indicates the importance of proper management of allergies while treatment planning for chronic rhinosinusitis. 
